# Effects of peripartal glucose precursor supplementation on lactation performance and metabolic health of primiparous and multiparous dairy cows

**DOI:** 10.5713/ab.22.0218

**Published:** 2022-11-14

**Authors:** Muhammad Uzair Akhtar, Hifzulrahman, Talat Naseer Pasha, Muhammad Avais, Nauman Khan, Ghazanfar Ali Chishti, Mubashar Ali, Muhammad Imran, Muhammad Naeem Tahir, Muhammad Naveed-ul-Haque

**Affiliations:** 1 Department of Animal Nutrition, University of Veterinary & Animal Sciences, Outfall road, Lahore 54000, Pakistan; 2 Department of Livestock Production, University of Veterinary & Animal Sciences, Outfall road, Lahore 54000, Pakistan; 3 Vice Chancellor of University of Education, College Road, Township, Lahore 54770, Pakistan; 4 Department of Veterinary Medicine, University of Veterinary & Animal Sciences, Outfall road, Lahore 54000, Pakistan; 5 Pakistan Holstein Pvt. Ltd., Risalpur 23200, Pakistan; 6 Department of Animal Nutrition, KBCMA College of Veterinary and Animal Sciences (Sub-campus University of Veterinary and Animal Sciences), Narowal 51600, Pakistan; 7 University College of Veterinary and Animal Sciences, the Islamia University of Bahawalpur, Bahawalpur 63100, Pakistan

**Keywords:** Glucogenic Precursor, β-Hydroxybutyrate, Multiparous Cow, Periparturient Cow, Primiparous Cow, Propylene Glycol

## Abstract

**Objective:**

Hyperketonemia remains a major metabolic issue of serious milk production and a major health concern in early lactation cows. Oral supplementation of glucose precursors (GP) can be used to prevent hyperketonemia in dairy cows. The objective of this study was to compare the beneficial effects of orally supplementing a mixture of GP on metabolic health indicators and milk production status of primiparous (PP) and multiparous (MP) dairy cows.

**Methods:**

Twenty-eight Holstein cows were blocked by expected date of parturition, previous lactation yield, and parity. The cows were randomly allocated to one of the four treatment groups (n = 7 cows/group) based on their parity and GP supplementation: i) PP cows fed basal diet only (PP-CON), ii) PP cows with oral supplementation of GP (PP-GP), iii) MP cows fed basal diet only (MP-CON), and iv) MP cows with oral supplementation of GP (MP-GP). Glucose precursor (glycoline liquid) was orally drenched (300 mL/d) in GP cows from 7 days prepartum through 7 days postpartum. Other than GP supplementation, all cows were fed similar pre- and postpartum basal diets.

**Results:**

In both pre- and postpartum periods, serum glucose concentration was increased, whereas β-hydroxybutyrate and free fatty acids were decreased in GP cows compared with the CON cows. Milk yield and milk components were statistically not different between GP and CON cows over the first 9 week of lactation. The yield of actual milk, energy-corrected milk, 63-days cumulative milk, colostrum yield, and calf birth weight remained higher in MP cows compared with PP cows.

**Conclusion:**

Oral drenching of GP around calving can be recommended to successfully improve the metabolic health and reduce the negative effects of hyperketonemia not only in MP but also in PP dairy cows.

## INTRODUCTION

Transition or periparturient period is a challenging 3 week (wk) pre- to 3 wk postpartum period in dairy cows [1]. Hyperketonemia is a common problem of early lactation and develops when supply of energy is not sufficient to satisfy the requirements for milk production. As a result, body reserves are used to supply free fatty acids (FFA). However, fat accumulation starts in liver when the supply of FFA exceeds the ability of liver to metabolize FFA, resulting in impaired liver function and postpartum hyperketonemia [1,2]. Rumination time is considered a promising indicator of metabolic health during the transition period and negative relation is reported between hyperketonemia and rumination time [3]. Despite the scientific knowledge about risk factors for hyperketonemia [4,5], the use of nutritional and management strategies [6] are not always beneficial to prevent hyperketonemia in dairy cows. As a result, 40% of the cows (up to 80% at some farms) experience hyperketonemia during early postpartum period with total cost of $375 and $256 per case of primiparous (PP) and multiparous (MP) cows, respectively [7]. This situation indicates that effective, safe, and practical preventive protocols for hyperketonemia still remain to be developed for dairy producers.

The supplementation of glucose precursors (GP) is one of the strategies used to prevent hyperketonemia at commercial dairy farms. Propylene glycol is supplemented as a precursor of ruminal propionate, which is rapidly absorbed in rumen for gluconeogenesis and being used to treat hyperketonemia since 1954 [8]. Similarly, glycerol having different route of conversion to glucose is also supplemented to reduce hyperketonemia. The basic objective of GP supplementation is to increase blood glucose, and decrease physiological imbalance in the body to minimize the production, health and eventually the economic losses. Glycerol is fermented to volatile fatty acids in the rumen and propylene glycol spares glucose for the udder by providing substrate for gluconeogenesis and inducing insulin resistance in peripheral tissues. The supplementation of GP is reported to increase blood glucose and decrease blood FFA and β-hydroxybutyrate (BHBA) concentrations [2,4]. Most of the previous studies have been conducted using various GPs as a powder top-dressing with different dose levels, treatment lengths, and lactational stages [9–11]. Typically, no metabolic response was observed when GPs were mixed in total mixed ration [8,9], whereas oral drenching of GP could be an effective way to reduce the effects of hyperketonemia [12,13]. The general trend of GP supplementation is to increase postpartum milk yield in dairy cows [2,8,13].

Replacement heifers are an essential investment and important for long-term sustainability of any dairy production system. At farms, little attention is paid to PP cows in terms of GP supplementation, assuming that PP cows experience less hyperketonemia compared with MP cows. Whereas it is observed that incidence of hyperketonemia is even more in PP than MP cows in some regions and prevalence could be up to 50% in PP cows [14]. Several authors observed the differences in energy metabolism of PP and MP dairy cows [15, 16]. However, as per authors’ knowledge, the differences in the effects of GP supplementation during the periparturient period on metabolic health, lactation performance, and rumination time has not been observed in PP versus MP dairy cows. We hypothesized that GP supplementation would be beneficial in both PP and MP cows by improving metabolic status due to increased glucose supply and decreased blood BHBA and FFA concentrations.

## MATERIALS AND METHODS

### Cows

This study was conducted at Pakistan Holstein Farm (34.08°N, 71.96°E, and 309 m altitude; Risalpur, Pakistan). Twenty-eight Holstein Friesian cows (12 PP and 16 multiparous [3rd parity]) dried at 60 days before expected date of parturition were enrolled in this study. The entire study was conducted under the protocols approved by the ethical committee for animal welfare at the University of Veterinary and Animal Sciences, Lahore (No. 7098). Primiparous cows were enrolled on the basis of (mean±standard deviation) body weight (BW; 695± 58 kg), body condition score (BCS; 3.27±0.07), and expected date of calving, whereas multiparous cows were enrolled on the basis of the expected date of calving, previous lactation yield (8,570±1,420 kg), BCS (3.39±0.12), and BW (720±80 kg).

### Experimental design and treatments

All the cows were fed a similar close-up diet during the prepartum period daily with half of the diet at 0730 and the remainder at 1830 h for *ad libitum* intake. The cows in each parity group received the treatments in a completely randomized block design in a 2×2 factorial arrangement. Primiparous and MP cows were randomly allocated to: i) control group (CON) fed basal diet only or ii) GP group fed basal diet + oral drenching of 300 mL/d of GP. The resulting four periparturient treatment groups were PP-CON, PP-GP, MP-CON, and MP-GP. Glycoline (Vitalac Co. Ltd., Carnoët, France) was used as a GP (contained 80% dry matter with 50% mono-propylene glycol and 40% other GPs including glycerol, sorbitol, and propionate). The supplementation of GP continued from 7 days prepartum to 7 days postpartum in the GP group. The enrolled cows received similar prepartum and postpartum basal diets. Ingredients and chemical composition of the treatment diets are presented in Table 1. Although cows were offered fixed amount of feed during the prepartum period (13.1 kg dry matter intake [DMI]/cow per day) for 100% intake, it was 145% of CNCPS predicted DMI. During the postpartum period, the total mixed ration was prepared daily (26.3 kg DMI/cow per day) with adjustment to allow 6% refusal. The basal diet was offered thrice daily at 0930, 1730, and 0130 h during the postpartum period. Postpartum data were collected over the 9 wk of lactation.

### Measurements, sampling, and analyses

Diets were fed as a total mixed ration after mixing in a mixer wagon (Dunker T1-80, Storti SPA, Italy) and all the ingredients were analyzed monthly for crude protein (method 984.13, N×6.25; Kjeldahl method), ether extract (method 920.39), and ash (method 942.05) contents following the official procedures of AOAC International [17]. The neutral detergent fiber (α-amylase + sodium sulfite treated filtration) and acid detergent fiber (sulfuric acid + cetyltrimethylammonium bromide treated filtration) were analyzed sequentially using the Ankom-2000 fiber analyzer (Ankom Technology Corp., Fairport, NY, USA).

Milking was done three times daily at 0900, 1700, and 0100 h (BouMatic LLC, Madison, WI, USA) and daily milk production was recorded electronically for each cow via the SmartEID ISO Ear Tag ID System (BouMatic LLC, USA). Milk samples for each cow were collected weekly during each milking and aliquots of each milking were composited in proportion to the respective milk yield to achieve the representative samples. Milk samples were analyzed with a Lactoscan milk analyzer (Lactoscan Farm Eco, Milkrotronic, Nova Zagora, Bulgaria) for fat, protein, and lactose contents.

Body condition score was measured once per wk at a 5-point scoring system and BW of the cows was measured weekly after morning milking and before feed distribution. Collection of blood samples was performed in the morning from the coccygeal blood vessels using evacuated tubes without preservative at day −7, −3, 3, 7, 14, 21 and weekly afterward relative to calving. Serum was separated following centrifugation at 2,000×g for 15 min and stored at −21°C for the later analysis of BHBA, FFA, glucose, blood urea nitrogen (BUN), and triglycerides (TG) using commercially available enzymatic kits as mentioned previously [18].

### Rumination time

All the cows were equipped with rumination monitoring loggers (SCR Engineers Ltd., Netanya, Israel) to observe the rumination time. These data loggers were positioned on the left side of neck with the help of a neck collar. Rumination produces a specific sound that is recorded by a microphone and processed by a microprocessor to determine the rumination time of the cows [19,20]. This system is comprised of rumination loggers, readers, and software to process the electronic records (Data Flow software; SCR Engineers Ltd., Israel). The neck collars communicate and send the data to data recorder with the help of an antenna. All the data are quantified in 2-h intervals and stored in the memory. The cows were provided a minimum 7 days of adaptation period after attachment of the neck collars.

### Calculations and statistical analysis

Energy-corrected milk (ECM), 3.4% protein-corrected milk (PCM), dietary non-fibrous carbohydrates, and changes in BW and BCS were calculated using the equations presented previously [18]. Before the statistical analysis, data collected on daily basis were converted to weekly means. Separate analyses were conducted for prepartum and postpartum 1 to 3 and 1 to 9 wk data. Repeated measures analysis was conducted for variables measured over time considering wk as a repeated measure. The GLIMMIX procedure of SAS (On-Demand for Academics; SAS, Institute Inc., Cary, NC, USA) was used for repeated measures analysis. Statistical model included fixed effects of parity (P), GP supplementation (G), T as time relative to calving, P×G interaction, P×T interaction, G×T interaction, and P×G×T interaction. The random error term used for all the models was cow and the covariance structure yielding the lowest Akaike’s information criterion was used. An autoregressive covariance structure was the best fit for this data using this methodology. Data are reported as least square means and results were declared significant at p≤0.05 and trend towards significance at 0.05<p≤0.10 using Tukey’s test.

## RESULTS

### Metabolic health

Effects of parity and periparturient GP supplementation on pre- and postpartum serum metabolites are presented in Table 2. Serum BHBA and FFA decreased, whereas glucose concentration increased in GP cows compared with CON cows during the prepartum period (p≤0.01). Serum BHBA and FFA concentrations decreased by 32.2% and 34.8% in GP versus CON cows over the 9 wk of lactation (p≤0.01). Postpartum serum glucose concentration increased in GP cows compared with CON cows (p = 0.03). Prepartum serum BHBA, FFA, glucose, TG, and BUN concentrations were similar between PP and MP groups (p>0.01). Postpartum serum BHBA concentration tended to increase in MP cows compared with PP cows over the 9 wk period (p = 0.07). Prepartum BHBA decreased (T, p = 0.05), FFA increased (T, p = 0.03), and BUN tended to increase (T, p = 0.08) as calving approached. Postpartum serum BHBA, FFA, and TG decreased, and BUN increased in all cows as lactation progressed (T, p≤0.05).

### Milk yield

Treatment means for postpartum milk yield are presented in Table 3. Glucose precursor supplementation had no effect on postpartum milk production and composition of PP and MP cows (p>0.10). Milk yield, ECM, and milk fat yield remained higher in MP cows compared with PP cows during the first 3 wk of lactation (p≤0.05). The yield of PCM (p = 0.10) tended to increase in MP cows compared with PP cows during the first 3 wk of lactation. Over the 9 wk period, milk yield, ECM, PCM, peak milk, and 63-days cumulative milk yield increased in MP cows compared with PP cows (p≤0.05). As lactation progressed, milk yield, ECM, PCM, milk lactose content and yield increased (T, p<0.10). The contents and yields of milk protein and fat decreased over the 9 wk of lactation (T, p<0.01).

### Calf birth weight and colostrum production

Effects of parity and periparturient GP supplementation on calf birth weight and colostrum production are presented in Table 4. Calves of MP cows had higher birth weight compared with PP cows, irrespective of the GP supplementation (p = 0.04). Calf average daily gain was not affected by periparturient GP supplementation to PP and MP cows (p>0.10). The GP supplementation had no effect on production and composition of colostrum (p>0.10). Colostrum yield and lactose content were higher in MP cows compared with PP cows (p≤0.05). Colostrum components and yields of energy corrected colostrum and protein corrected colostrum were similar between MP and PP cows (p>0.10).

### BCS and BW

Results on periparturient BCS, and BW are presented in Table 5. The supplementation of GP had no effect on pre- and postpartum BW and BCS of MP and PP cows (p>0.10). The BCS of MP cows was greater than PP cows during the prepartum period (p = 0.02). The BCS, BW, and BCS change were not different between MP and PP cows (p>0.10). The postpartum BW change was higher in MP cows compared with PP cows (p<0.01). The MP cows tended to lose higher BW compared with the PP cows as lactation progressed over the 9 wk period (P×T interaction, p = 0.08). Postpartum BW and BCS decreased in all cows as lactation progressed (T, p<0.01).

### Rumination time

Rumination time of different treatment groups are presented in Table 6. The GP cows tended to have increased rumination time from prepartum (p = 0.10) through calving day (p = 0.08) and first 3 wk postpartum period (p = 0.07). Rumination time was greater in MP cows compared with PP cows on calving day and during the pre- and postpartum periods (p≤0.05). Rumination time decreased in all cows as calving approached (T, p<0.01) and this decrease in rumination time was greater in PP versus MP cows (P×T interaction, p = 0.01) and CON versus GP cows (G×T interaction, p<0.01). Rumination time increased in all cows as lactation progressed (T, p<0.01).

## DISCUSSION

The primary focus of this study was to observe the effects of oral drenching of GP (300 mL/d) from 7 days prepartum through 7 days postpartum period in Holstein dairy cows of different parity groups. Pre- and postpartum basal diets were similar in all the treatment groups during the experimental period.

### Glucose precursor decreased serum BHBA and FFA while increased glucose concentration

**Figure 1:**
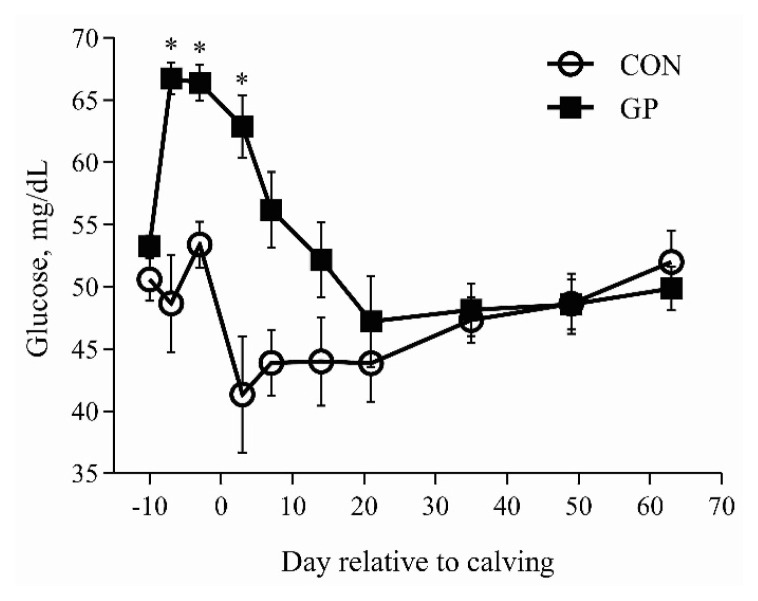
Least square means for pre- and postpartum serum glucose concentration, mg/dL. Treatment abbreviations: CON = cows fed basal diet only and GP = basal diet + oral drenching of 300 mL/d glucose precursor supplementation from 7 days prepartum to 7 days postpartum. Significant differences between treatments on specific days (p<0.05) are denoted by the asterisk (*). All the cows were fed similar pre- and postpartum diets.

**Figure 2:**
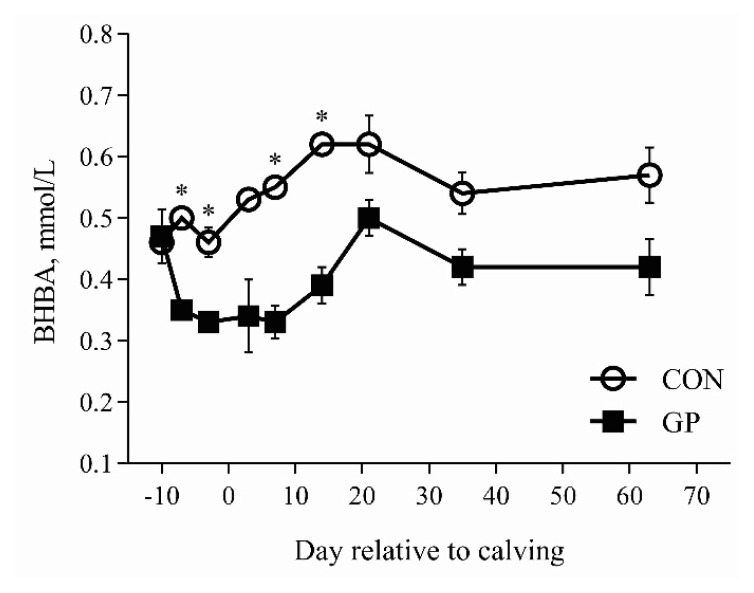
Least square means for pre- and postpartum serum β-hydroxybutyrate (BHBA) concentration, mmol/L. Treatment abbreviations: CON = cows fed basal diet only and GP = basal diet + oral drenching of 300 mL/d glucose precursor supplementation from 7 days prepartum to 7 days postpartum. Significant differences between treatments on specific days (p<0.05) are denoted by the asterisk (*). All the cows were fed similar pre- and postpartum diets.

The concentration of serum glucose increased and serum BHBA and FFA decreased with GP supplementation in agreement with literature studies [8,10,13]. Propylene glycol was the primary component of the GP used in this study, which increases concentration of blood glucose and decreased concentrations of blood BHBA and FFA by improving the molar proportion of ruminal propionate and hepatic gluconeogenesis [2]. The administration method is also important and GP supplementation through oral drenching is considered the most effective. McArt et al [12] reported that 300 mL/d of propylene glycol supplementation increased the chances of resolving sub-clinical hyperketonemia by 1.50 times, and 0.54 time less likely to develop clinical hyperketonemia than control cows. Typically, the increase in postpartum BHBA and FFA occurs with increased postpartum BCS loss. Interestingly, the decrease in postpartum BHBA and FFA with an increase in glucose concentration happened without any change in postpartum BCS in our study. However, it was confirmed previously that postpartum decrease in BHBA and FFA is a possible response to GP supplementation without change in BCS [11]. The changes in serum glucose and BHBA concentrations during the periparturient period are plotted in Figure 1 and 2. The supplementation of GP is typically practiced only in MP cows. Keeping in view the positive response of PP cows in our study, it is possible that PP cows just require an optimum dose supplemented through a proper method for a specific time, which requires further investigation. The postpartum BHBA concentration tended to remain lower in PP cows compared with the MP cows in this study. Although prepartum BCS was also lower in PP versus MP cows in our study, it is observed previously that over-conditioned PP cows are less prone to postpartum lipolysis and during the postpartum period contrary to the MP cows [16].

### Supplementation of GP numerically increased milk yield of PP and MP cows

Although milk yield was not increased with GP supplementation, a numeric increase of 2.55 kg/d in GP versus CON cows was observed in this study. Several authors reported no improvement in milk production with GP supplementation [21–23]. Contrarily, a significant increase in milk yield with GP supplementation was observed when cows had clinical or subclinical ketosis [12,24]. The inconsistency in the results of various studies might be due to the dose of GP or use of GP in cows with different metabolic status [2]. Interestingly, the numeric increase in 63-days cumulative milk yield was 107 kg in MP-GP versus MP-CON cows, whereas this increase was 267 kg in PP-GP versus PP-CON cows, which indicates that PP cows benefitted even more from decreased FFA and BHBA due to GP supplementation [25]. However, there could be two possible reasons of no statistical difference in milk production of GP versus CON cows in our study. First, low incidence of hyperketonemia in well-managed herd with good nutritional program (indicated by low BHBA even in CON cows) might override the possible clear effects of GP [21,23]. Second, increased energy supply due to GP supplementation might have been partially used to reduce the postpartum BW loss [22]. Although lactating cows were fed for *ad libitum* intake, a limitation of this experiment is that individual feed intake of the enrolled cows was not measured, which could better explain the observed results of milk production.

### Higher milk yield in MP versus PP cows

Milk and colostrum yields were greater in MP cows compared with the PP cows in agreement with several previous studies [15,26]. This might be explained by the fact that PP cows are typically smaller, eat less, and their milk production is low as compare to multiparous cows [26]. The higher milk yield of MP cows can also be attributed to more developed mammary gland compared with the PP cows [27].

### The PP cows gave birth to smaller calves than MP cows

Primiparous cows gave birth to smaller calves compared with multiparous cows. The difference in calf birth weight of PP and multiparous cows was 3.60 kg in this study, which is close to previously reported parity differences of 3.50 and 3.43 kg [28,29]. The reason could be the partitioning of nutrients for growth of the PP cows compared with MP cows also indicated by lower BW loss in PP cows compared with MP cows during the postpartum period.

### Rumination time tended to increase in GP versus CON cows

Rumination time is monitored as a useful indicator of metabolic health and early detection of subclinical diseases before the appearance of clinical signs [3]. A decrease in rumination time was experienced by both PP and multiparous cows around calving in this study irrespective of the parity and GP supplementation. Similar findings are reported in literature studies indicating that this decrease in rumination time before calving could be associated with the decrease in feed intake, which typically decreases before calving and increases after calving [30]. As per authors’ knowledge, this is the first study reporting rumination time of PP and multiparous cows supplemented with GP. As rumination time is closely associated with dietary neutral detergent fiber intake and particle size [30], supplementation level of GP used in this study might have minor effects on fiber intake. Nevertheless, an increase of >10% in rumination time of GP cows during the prepartum, postpartum, and calving day is indicative of better metabolic health compared with CON cows in this study [3].

## CONCLUSION

The concentration of glucose increased, whereas BHBA and FFA decreased in GP cows compared with the CON cows during the pre- and postpartum periods. Multiparous cows had higher milk production, ECM, 63-days cumulative milk yield, and calf birth weight compared with PP cows. The results of our study indicate that oral drenching of GP from 7 days prepartum through 7 days postpartum is beneficial in both PP and multiparous dairy cows.

## Figures and Tables

**Table 1:** Composition of basal diets fed during pre- and postpartum periods

Item	Prepartum	Postpartum
Ingredients (% of DM)		
Corn silage	35.6	32.8
Rhodes grass hay	6.58	6.53
Alfalfa haylage	-	2.73
Wheat straw	21.1	2.10
Corn grain	13.0	23.3
Soybean meal	10.5	12.2
Canola meal	7.02	8.70
Corn gluten meal 60%	-	2.81
Sugarcane molasses	-	4.16
Calcium carbonate	1.52	0.57
Di-calcium phosphate	0.23	0.19
Sodium bicarbonate	-	0.66
Magnesium sulfate	0.38	-
Sodium chloride	0.38	0.19
Mineral premix^1)^	1.14	0.86
Close-up premix^2)^	1.52	-
Urea	-	0.19
Bypass fat^3)^	0.74	1.84
Toxin binder (Bentonite)	0.15	0.08
Yeast^4)^	-	0.03
Mintrex^5)^	-	0.02
Vitamin E premix^6)^	0.02	0.01
Methionine^7)^	0.07	0.03
Analyzed nutrient composition		
% DM	53.7	54.2
CP (% of DM)	13.6	17.1
NDF (% of DM)	41.4	29.1
ADF (% of DM)	26.3	16.7
NFC (% of DM)	33.5	43.6
Ether extract (% of DM)	3.54	4.85
Predicted nutritive values^8)^		
MP (g/kg of DM)	87.3	120
RUP (% CP)	30.0	41.3
RDP (% CP)	70.0	58.7
ME (Mcal/kg of DM)	2.25	2.75
NEL (Mcal/kg of DM)	1.45	1.77
DCAD (mEq/kg)	−100	210

DM, dry matter; CP, crude protein; NDF, neutral detergent fiber; ADF, acid detergent fiber; NFC, non-fibrous carbohydrates; MP, metabolizable protein; RUP, rumen undegradable protein; RDP, rumen degradable protein; ME, metabolizable energy; NEL, net energy for lactation; DCAD, dietary cation anion difference.

1) Contained 7.00% Ca, 8.00% P, 1.50% Mg, 2.60% K, 2.60% S, 3.00% Na, 4.70% Cl, 0.50% Fe, 1.70% Zn, 0.16% Cu, 0.18% Mn, 0.004% Se, 0.001% Co, 0.01% I, 600 IU/g of vitamin A, 180 IU/g of vitamin D, and 4.20 IU/g of vitamin E.

2) Contained 10.5% Ca, 8.50% S, and 33.0% Cl.

3) FATELAC, UM Enterprises, Pakistan. Rumen protected fat 84%.

4) Saccharomyces cerevisiae fermentation product (Amino yeast, Specialty Biotec Co., Ltd., Thailand).

5) MINTREX dairy, Novus International Inc., USA. Contained 10.9% Ca, 0.33% P, 0.81% Mg, 1.08% K, 1.48% S, 0.02% Na, 0.03% Cl, 7.20% Zn, 1.29% Cu, 2.00% Mn, 0.03% Se, and 0.13% Co.

6) Contained 500,000 IU/kg of vitamin E.

7) MetaSmart dry (Adisseo Inc.).

8) Predicted using CNCPS evaluation.

**Table 2:** Effects of periparturient glucose precursor supplementation and parity on blood metabolites

Item	Periparturient treatment^1)^				SEM	p-value^2)^						

	CON		GP									
		
	PP	MP	PP	MP		G	P	T	G×P	G×T	P×T	G×P×T
Prepartum												
BHBA (mmol/L)	0.48	0.47	0.33	0.34	0.029	<0.01	0.85	0.05	0.78	0.25	0.12	0.09
FFA (mEq/L)	0.32	0.29	0.19	0.19	0.056	0.05	0.72	0.03	0.82	0.63	0.31	0.47
TG (mg/dL)	19.4	20.2	18.9	20.4	1.39	0.89	0.37	0.49	0.83	0.61	0.97	0.42
Glucose (mg/dL)	46.1	54.1	67.1	65.6	3.36	<0.01	0.29	0.29	0.13	0.13	0.25	0.97
BUN (mg/dL)	11.7	11.4	10.0	11.9	0.95	0.49	0.38	0.08	0.21	0.34	0.59	0.17
Postpartum												
wk 1 to 3												
BHBA (mmol/L)	0.54	0.62	0.36	0.42	0.042	<0.01	0.10	<0.01	0.76	0.37	0.12	0.48
FFA (mEq/L)	0.32	0.40	0.23	0.21	0.040	0.01	0.42	0.30	0.27	0.81	0.86	0.93
TG (mg/dL)	17.5	18.4	18.7	18.2	0.61	0.37	0.71	0.85	0.28	0.92	0.53	0.48
Glucose (mg/dL)	38.6	49.1	53.3	55.7	3.29	<0.01	0.06	0.25	0.22	0.08	0.64	0.77
BUN (mg/dL)	12.8	13.2	12.7	12.7	1.04	0.75	0.81	0.35	0.83	0.40	0.20	0.23
wk 1 to 9												
BHBA (mmol/L)	0.52	0.66	0.38	0.42	0.044	<0.01	0.07	<0.01	0.24	0.29	0.17	0.13
FFA (mEq/L)	0.30	0.36	0.21	0.22	0.037	0.01	0.36	0.03	0.43	0.29	0.31	0.48
TG(mg/dL)	16.8	17.9	18.2	18.0	0.41	0.12	0.29	0.05	0.14	0.69	0.49	0.57
Glucose (mg/dL)	42.8	49.3	51.6	53.5	2.71	0.03	0.13	0.34	0.40	<0.01	0.24	0.66
BUN (mg/dL)	13.4	13.7	13.0	14.0	1.212	0.99	0.58	<0.01	0.76	0.50	0.29	0.22

CON, control; GP, glucose precursor supplementation; PP, primiparous; MP, multiparous; SEM, standard error of the mean; BHBA, β-hydroxybutyrate; FFA, free fatty acids; TG, triglycerides; BUN, blood urea nitrogen.

1) PP and MP cows received: i) basal diet only = CON or ii) basal diet + oral supplementation of 300 mL/d glucose precursor from 7 days prepartum to 7 days postpartum = GP.

2) Probability of treatment effects: G = effects of glucose precursor supplementation, CON versus GP; P = effects of parity, PP versus MP; T = effects of time relative to calving.

**Table 3:** Effects of periparturient glucose precursor supplementation and parity on milk production and composition

Item	Periparturient treatment^1)^				SEM	p-value^2)^						

	CON		GP									
		
	PP	MP	PP	MP		G	P	T	G×P	G×T	P×T	G×P×T
wk 1 to 3												
Actual milk (kg/d)	26.8	33.6	28.6	34.8	3.09	0.59	0.02	<0.01	0.91	0.53	0.97	0.50
ECM (kg/d)	31.1	38.0	30.2	39.5	4.09	0.94	0.05	<0.01	0.73	0.27	0.30	0.26
3.4% PCM (kg/d)	24.8	30.8	26.2	30.4	3.22	0.88	0.10	<0.01	0.74	0.28	0.98	0.40
Fat (%)	4.11	4.20	3.90	4.35	0.256	0.89	0.26	0.67	0.44	0.92	0.42	0.13
Fat (g/d)	1,097	1,416	1,113	1,525	171.0	0.69	0.04	0.01	0.76	0.78	0.32	0.12
Protein (%)	2.95	3.05	2.92	2.99	0.053	0.32	0.11	<0.01	0.79	0.24	0.76	0.36
Protein (g/d)	795	1037	849	1041	128.3	0.81	0.11	<0.01	0.83	0.23	0.85	0.17
Lactose (%)	4.22	4.02	4.11	4.09	0.077	0.71	0.14	0.26	0.22	0.84	0.38	0.13
Lactose (g/d)	1,143	1,366	1,181	1,433	160.6	0.72	0.15	<0.01	0.92	0.51	0.85	0.11
wk 1 to 9												
Actual milk (kg/d)	32.9	41.4	36.0	43.4	3.00	0.34	<0.01	<0.01	0.84	0.77	0.34	0.60
ECM (kg/d)	35.2	43.5	37.7	45.7	3.96	0.52	0.03	<0.01	0.97	0.38	0.57	0.61
3.4% PCM (kg/d)	28.7	35.4	32.2	37.1	2.94	0.33	0.04	<0.01	0.73	0.40	0.60	0.74
Fat (%)	3.73	3.80	3.50	3.71	0.205	0.38	0.43	<0.01	0.71	0.43	0.71	0.45
Fat (g/d)	1,235	1,573	1,283	1,624	196.9	0.79	0.10	<0.01	0.99	0.62	0.82	0.40
Protein (%)	2.85	2.88	2.84	2.85	0.048	0.61	0.62	<0.01	0.76	0.23	0.16	0.66
Protein (g/d)	951	1,198	1,039	1,254	145.1	0.58	0.08	<0.01	0.90	0.21	0.42	0.60
Lactose (%)	4.27	4.23	4.29	4.26	0.077	0.77	0.58	<0.01	0.96	0.92	0.23	0.18
Lactose (g/d)	1,403	1,769	1,567	1,849	235.4	0.56	0.13	<0.01	0.84	0.80	0.57	0.53
Peak milk (kg/d)	43.3	54.6	49.0	55.7	3.51	0.25	<0.01	-	0.42	-	-	-
63-day cumulative milk yield (kg)	1,992	2,590	2,254	2,697	240.3	0.36	0.02	-	0.69	-	-	-

CON, control; GP, glucose precursor supplementation; PP, primiparous; MP, multiparous; SEM, standard error of the mean; Wk, week; ECM, energy corrected milk; PCM, protein corrected milk.

1) PP and MP cows received: i) basal diet = CON or ii) basal diet + oral supplementation of 300 mL/d glucose precursor from 7 days prepartum to 7 days postpartum = GP.

2) Probability of treatment effects: G = effects of glucose precursor, CON versus GP; P = effects of parity, PP versus MP; T = effects of time relative to calving.

**Table 4:** Effects of periparturient glucose precursor supplementation and parity on colostrum yield and composition

Item	Periparturient treatment^1)^				SEM	p-value^2)^		

	CON		GP					
		
	PP	MP	PP	MP		G	P	G×P
Calf birth weight (kg)	36.8	39.2	33.3	38.1	1.937	0.18	0.04	0.49
Calf daily weight gain (kg)	0.55	0.53	0.50	0.60	0.047	0.79	0.37	0.23
Colostrum yield								
Actual (kg)	5.33	7.75	3.67	6.56	1.419	0.28	0.05	0.85
Energy corrected (kg)	11.2	13.9	5.96	10.4	3.13	0.14	0.23	0.76
3.4% Protein corrected (kg)	10.7	13.0	5.50	13.1	3.58	0.44	0.15	0.43
Fat (%)	7.16	7.65	6.24	6.20	1.131	0.27	0.83	0.79
Protein (%)	5.31	5.29	4.72	6.73	0.663	0.49	0.12	0.11
Lactose (%)	7.37	9.27	6.55	11.5	0.962	0.44	<0.01	0.11
Colostrum IgG (mg/mL)	65.4	59.7	65.5	60.9	2.819	0.79	0.04	0.82

CON, control; GP, glucose precursor supplementation; PP, primiparous; MP, multiparous; SEM, standard error of the mean.

1) PP and MP cows received: i) basal diet only = CON or ii) basal diet + oral supplementation of 300 mL/d glucose precursor from 7 days prepartum to 7 days postpartum = GP.

2) Probability of treatment effects: G = effects of glucose precursor supplementation, CON versus GP; P = effects of parity, PP versus MP.

**Table 5:** Effects of periparturient glucose precursor supplementation and parity on BW and BCS

Item	Periparturient treatment^1)^				SEM	p-value^2)^						

	CON		GP									
		
	PP	MP	PP	MP		G	P	T	G×P	G×T	P×T	G×P×T
Prepartum												
BCS	3.30	3.40	3.25	3.38	0.050	0.51	0.02	-	0.67	-	-	-
BW (kg)	692	741	698	707	39.2	0.68	0.41	-	0.56	-	-	-
Postpartum												
BCS	2.97	3.00	2.95	3.04	0.051	0.86	0.26	<0.01	0.56	0.88	0.20	0.59
BW (kg)	603	642	608	627	35.2	0.87	0.38	<0.01	0.77	0.56	0.08	0.63
BCS change	−0.20	−0.30	−0.16	−0.19	0.062	0.23	0.27	-	0.53	-	-	-
BW change (kg)	−26.6	−39.2	−18.2	−35.3	4.56	0.14	<0.01	-	0.57	-	-	-

BW, body weight; BCS, body condition score; CON, control; GP, glucose precursor supplementation; PP, primiparous; MP, multiparous; SEM, standard error of the mean.

1) PP and MP cows received: i) basal diet only = CON or ii) basal diet + oral supplementation of 300 mL/d glucose precursor from 7 days prepartum to 7 days postpartum = GP.

2) Probability of treatment effects: G = effects of glucose precursor supplementation, CON versus GP; P = effects of parity, PP versus MP; T = effects of time relative to calving.

**Table 6:** Effects of periparturient glucose precursor supplementation and parity on rumination time

Item	Periparturient treatment^1)^				SEM	p-value^2)^						

	CON		GP									
		
	PP	MP	PP	MP		G	P	T	G×P	G×T	P×T	G×P×T
Prepartum	337	428	374	488	28.6	0.10	<0.01	<0.01	0.67	<0.01	0.01	0.95
Calving day	182	266	202	315	18.5	0.08	<0.01	-	0.42	-	-	-
Postpartum												
wk 1 to 3	378	435	430	471	24.8	0.07	0.05	<0.01	0.75	0.96	0.74	0.91
wk 1 to 9	411	488	444	517	21.0	0.13	<0.01	<0.01	0.91	0.77	0.20	0.78

CON, control; GP, glucose precursor supplementation; PP, primiparous; MP, multiparous; SEM, standard error of the mean.

1) PP and MP cows received: i) basal diet only = CON or ii) basal diet + oral supplementation of 300 mL/d glucose precursor from 7 days prepartum to 7 days postpartum = GP.

2) Probability of treatment effects: G = effects of glucose precursor supplementation, CON versus GP; P = effects of parity, PP versus MP; T = effects of time relative to calving.
